# Study of some of quality process indicators of hospital emergency services for Victims of traffic accidents in Imam Khomeini hospital

**Published:** 2019-08

**Authors:** Sima Marzban, Abbas Daneshkohan, Marziye Najafi

**Affiliations:** ^*a*^School of Health, Shahid Beheshti University of Medical Sciences, Tehran, Iran.; ^*b*^School of Public Health, Tehran University of Medical Sciences, Tehran, Iran.

## Abstract

**Background:**

Every year in the world 2.1 million people are killed in road accidents, and more than 50 million are either injured or disabled. The way to enter the hospital for the Victims of traffic accidents in order to receive treatment in Emergency Department (ED). The low quality of the offered services in this ward leads to a decrease in the patient’s satisfaction, an increase in the number of deaths and handicaps. So we decided to evaluate the quality of the procedure of the offered treatments to the Victims of traffic accidents based on indicators derived from the indicators of quality evaluation of AHRQ services of ED in Imam Khomeini hospital complex.

**Methods:**

The statistical society in this study consists of those who were injured in traffic indicators and brought to the emergency section of Imam Khomeini hospital. The indicators in mind were taken from the repertoire of AHRQ indicators which evaluate quality aspects (effectiveness, timeliness, safety, efficiency, patient-centeredness), and whose validity and reliability have been proven in Romano’s study, albeit with minute alterations. The findings were given to SPSS18 software and were analyzed using descriptive statistics and statistical tests.

**Results:**

According to the findings of this study, 42% of the injured experienced hemorrhage which were controlled successfully, and the average time for controlling it found to be about 3 minutes. The starting time for preliminary examinations was reported at 3.37 minutes. Immediate sonography was done for 100% of the patients with abdominal problems. 100% of the intubation operations on patients were done correctly. 96.49% of the patients were prescribed sedatives.

**Conclusions:**

The indicators under study show an appropriate state in the processes of ED of the hospital investigated. Regarding the importance of managing and controlling pain in urgent patients, the hospital needs to compile processes for measuring and controlling pain. The investigation of the processes of ED is recommended to be done with the indicators of structure and the results.

**Keywords:**

Emergency Services, Quality process indicators, Quality, Victims of traffic accidents

